# Microstructural remodeling in the post-infarct porcine heart measured by diffusion tensor MRI and T1-weighted late gadolinium enhancement MRI

**DOI:** 10.1186/1532-429X-14-S1-P66

**Published:** 2012-02-01

**Authors:** Geoffrey L Kung, Olujimi Ajijola, Rafael J Ramirez, Jin Kyu Gahm, Wei Zhou, N Wisniewski, Aman Mahajan, Alan Garfinkel, Kalyaman Shivkumar, Daniel Ennis

**Affiliations:** 1 Department of Radiological Sciences, University of California, Los Angeles, Los Angeles, CA, USA; 2 Biomedical Engineering IDP, University of California, Los Angeles, Los Angeles, CA, USA; 3 Cardiac Arrhythmia Center, David Geffen School of Medicine, University of California, Los Angeles, Los Angeles, CA, USA; 4 Computer Science Department, University of California, Los Angeles, Los Angeles, CA, USA; 5 Cardiac Anesthesia, Department of Anesthesia, David Geffen School of Medicine, University of California, Los Angeles, Los Angeles, CA, USA; 6 Cardiovascular Research Laboratory, Department of Cardiology, David Geffen School of Medicine, University of California, Los Angeles, Los Angeles, CA, USA

## Summary

The objective of this study was to quantify microstructural remodeling in peri-infarcted and infarcted porcine myocardium using diffusion tensor MRI (DT-MRI) for the first time. High resolution ex vivo late gadolinium enhanced (LGE) MRI was used to segment the DT-MRI data into normal, peri-infarct and infarcted myocardium. LGE-MRI based segmentation produces regions with significantly different microstructural remodeling.

## Background

T1-weighted late gadolinium enhanced (LGE) magnetic resonance imaging (MRI) is recognized as the “gold standard” for MRI based myocardial infarct mapping [1]. It is unclear how variations in LGE signal intensity relate to microstructural remodeling. Diffusion tensor magnetic resonance imaging (DT-MRI) enables 3D evaluation of soft tissue microstructure. Specifically, DT invariants provide a basis for evaluating changes in the trace (TR, magnitude-of-isotropic-diffusion), fractional anisotropy (FA, magnitude-of-anisotropy), and the tensor mode (MD, type-of-anisotropy) as a consequence of remodeling [2]. Previous DT-MRI studies of microstructural remodeling in post-infarct myocardium have not used LGE to segment the remote, peri-infarcted, and infarcted myocardium [3,4]. The objective of this study was to use high resolution ex vivo LGE MRI of post-infarct porcine hearts to segment remote, peri-infarcted and infarcted myocardium and subsequently use this segmentation to quantify microstructural remodeling in peri-infarcted and infarcted myocardium using DT-MRI for the first time.

## Methods

Antero-septal infarctions in adult female porcine hearts (N=3) were achieved via micro-bead injection distal to the mid left anterior descending coronary artery. After 8-weeks, Gd-DTPA was injected (0.1mmol/kg) and allowed to circulate for 15 minutes before euthanizing. Normal adult porcine hearts (M=3) served as controls. The hearts were excised and LGE MRI (0.33x0.33x0.50mm resolution) began within 2 hours of sacrifice. Immediately afterwards, co-registered DT-MRI (1x1x3mm resolution) was performed. Myocardial voxels were segmented as normal, peri-infarct or infarct based on signal intensity (SI) thresholds of the LGE images for each heart. We defined bootstrapped histograms and medians with 95% confidence intervals (95%-CIs) of each DT invariant in order to make statistical comparisons of non-Gaussian datasets tractable. Remote myocardium in infarcted hearts was also compared to myocardium of control hearts.

## Results

**Figure 1:**
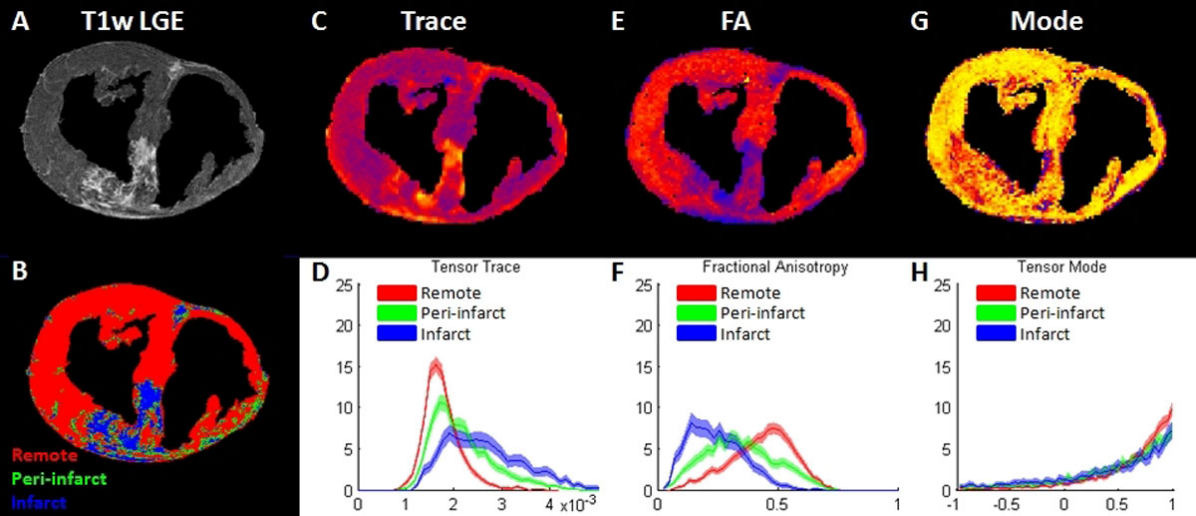
(A) Short-axis T1w LGE image depicting enhancement of the infarct. (B) Segmentation of the T1w LGE slice into remote, peri-infarcted and infarcted myocardium using LGE SI thresholds. Trace map (C) and whole heart segmented bootstrapped histograms (D); FA map (E) and whole heart segmented bootstrapped histograms (F); mode map (G) and whole heart segmented bootstrapped histograms (H).

DT invariant medians and 95%-CIs for segmented myocardium in each heart are listed in Table 1. Invariants in control hearts were similar to remote myocardium in infarcted hearts (Table 1). Intra-heart statistical differences between segmented myocardium were significant. Figure 1 depicts invariant maps for a short axis slice of one heart accompanied by bootstrapped histograms with 95%-CIs for invariant data for the whole heart. LGE and remote/infarct/peri-infarct segmentation for the same slice are also shown.

**Table 1:** Tensor invariant medians and 95%-CIs for normal, remote, peri-infarcted, and infarcted myocardium

Heart/region	**Median trace (mm**^2^** /s)**	**95%-CI of median trace (mm**^2 ^**/s)**	Median FA	95%-CI of median FA	Median mode	95%-CI of median mode
Control 1: Normal	0.0017	[0.00170, 0.00174]	0.46	[0.453, 0.477]	0.76	[0.744, 0.781]
Control 2: Normal	0.0014	[0.00140, 0.00145]	0.45	[0.437, 0.455]	0.72	[0.700, 0.743]
Control 3: Normal	0.0017	[0.00166, 0.00170]	0.47	[0.462, 0.478]	0.71	[0.691, 0.740]
Infarct 1: Remote	0.0017	[0.00174, 0.00176]	0.44	[0.435, 0.444]	0.71	[0.699, 0.723]
Infarct 2: Remote	0.0017	[0.00170, 0.00172]	0.45	[0.442, 0.450]	0.73	[0.712, 0.733]
Infarct 3: Remote	0.0020	[0.00194, 0.00196]	0.43	[0.430, 0.437]	0.72	[0.709, 0.726]
Infarct 1: Peri-infarct	0.0019	[0.00186, 0.00190]	0.39	[0.380, 0.391]	0.62	[0.605, 0.643]
Infarct 2: Peri-infarct	0.0020	[0.00200, 0.00205]	0.34	[0.332, 0.347]	0.62	[0.605, 0.642]
Infarct 3: Peri-infarct	0.0023	[0.00225, 0.00230]	0.34	[0.334, 0.347]	0.62	[0.607, 0.645]
Infarct 1: Infarct	0.0023	[0.00223, 0.00234]	0.28	[0.264, 0.288]	0.40	[0.343, 0.454]
Infarct 2: Infarct	0.0025	[0.00234, 0.00254]	0.23	[0.225, 0.240]	0.57	[0.539, 0.586]
Infarct 3: Infarct	0.0025	[0.00247, 0.00259]	0.27	[0.265, 0.283]	0.55	[0.502, 0.572]

## Conclusions

LGE segmentation of DT-MRI data identifies regions of statistically significant microstructural remodeling in peri-infarcted and infarcted myocardium. Improved LGE segmentation methods hold promise for identifying regions of microstructural remodeling when DT-MRI is not available.

## Funding

Mahajan R01-HL084261; Shivkumar R01-HL084261 and R01-HL067647; Garfinkel P01-HL78931 (Core).
